# Draft genome sequence of wild *Prunus yedoensis* reveals massive inter-specific hybridization between sympatric flowering cherries

**DOI:** 10.1186/s13059-018-1497-y

**Published:** 2018-09-04

**Authors:** Seunghoon Baek, Kyung Choi, Goon-Bo Kim, Hee-Ju Yu, Ara Cho, Hoyeol Jang, Changkyun Kim, Hyuk-Jin Kim, Kae Sun Chang, Joo-Hwan Kim, Jeong-Hwan Mun

**Affiliations:** 1 Department of Bioscience and Bioinformatics, Myongji University, Yongin, 17058 Korea; 2 Korea National Arboretum, Pocheon, 11186 Korea; 3 Department of Life Science, The Catholic University of Korea, Bucheon, 14662 Korea; 4 Department of Life Science, Gachon University, Seongnam, 13120 Korea

**Keywords:** Flowering *Prunus*, Hybrid genome, Long-read sequencing, Sequence phase, S-locus haplotype

## Abstract

**Background:**

Hybridization is an important evolutionary process that results in increased plant diversity. Flowering *Prunus* includes popular cherry species that are appreciated worldwide for their flowers. The ornamental characteristics were acquired both naturally and through artificially hybridizing species with heterozygous genomes. Therefore, the genome of hybrid flowering *Prunus* presents important challenges both in plant genomics and evolutionary biology.

**Results:**

We use long reads to sequence and analyze the highly heterozygous genome of wild *Prunus yedoensis*. The genome assembly covers > 93% of the gene space; annotation identified 41,294 protein-coding genes. Comparative analysis of the genome with 16 accessions of six related taxa shows that 41% of the genes were assigned into the maternal or paternal state. This indicates that wild *P. yedoensis* is an F1 hybrid originating from a cross between maternal *P. pendula* f. *ascendens* and paternal *P*. *jamasakura*, and it can be clearly distinguished from its confusing taxon, Yoshino cherry. A focused analysis of the S-locus haplotypes of closely related taxa distributed in a sympatric natural habitat suggests that reduced restriction of inter-specific hybridization due to strong gametophytic self-incompatibility is likely to promote complex hybridization of wild *Prunus* species and the development of a hybrid swarm.

**Conclusions:**

We report the draft genome assembly of a natural hybrid *Prunus* species using long-read sequencing and sequence phasing. Based on a comprehensive comparative genome analysis with related taxa, it appears that cross-species hybridization in sympatric habitats is an ongoing process that facilitates the diversification of flowering *Prunus*.

**Electronic supplementary material:**

The online version of this article (10.1186/s13059-018-1497-y) contains supplementary material, which is available to authorized users.

## Background

Over the past several decades, genome analyses of diverse plant species have revealed that almost all plant genomes have experienced polyploidy events during their evolutionary history, suggesting that polyploidy has played an important role in plant diversification and speciation (reviewed in [1]). Plant speciation that arises from polyploidy occurs via genome doubling within a species or through hybridization, either between closely related populations of the same species (autopolyploidy) or, more commonly, inter-specific or intergeneric hybridization (allopolyploidy). In both hybridization processes, generation of a heterozygous genome by hybridization can be a potential source of new species; the heterozygous filial generations may show different levels of heterosis or inbreeding depression due to variation between the homologous chromosomes. There are many examples of natural hybrid species reported from a wide range of monocots and dicots. In addition, inter-specific hybrid plants have been developed by breeding programs for agricultural or commercial purposes. Therefore, hybridization has been considered a creative force of evolution in plants [2]. Additionally, the sequencing and assembly of the genomes of hybrid plants presents an important challenge.

Plant genome sequencing has been facilitated by the introduction of next-generation sequencing (NGS) technologies that enable individual research groups to sequence and assemble the entire genome of interest. The sequencing and assembly of the genomes of plants, especially crop species, has typically been performed with homozygous or inbred lines using short-read sequencing; the resulting assemblies represent the homozygous haploid genome. In contrast, assemblies of heterozygous individuals, including natural tree species, require accurate handling of sequence reads to reconstruct the separate chromosome sets. For this reason, heterozygous plant genomes still pose considerable challenges in genome sequencing and assembly. To overcome the weaknesses of short-read assemblies, which cannot resolve a mixture of highly similar sequences such as duplicated genes or repetitive transposons [3], long-read sequencing technology, such as PacBio sequencing, is a good choice because long-read data can be phased into individual chromosomes during assembly of the reads [4]. For example, an *Arabidopsis* F1 hybrid genome and a heterozygous grapevine accession genome were successfully de novo assembled into haplotigs using a FLACON assembler and PacBio reads [5]. Therefore, long-read sequencing technology combined with relevant assembly algorithms could help the assembly of the heterozygous genomes derived from hybridization.

Rosaceae is a flowering plant family consisting of approximately 3000 species in 90 genera and large number of inter-specific and intergeneric hybrids [6]. The rose family includes diverse plant species such as herbs, shrubs, and trees that are widespread in northern temperate regions. A number of species are economically important as food crops that produce fruits and nuts. Due to their economic and agronomic importance, the genomes of several Rosaceae fruit crop species have been sequenced, including the domesticated apple [7], sweet cherry [8], Chinese plum [9], peach [10], pear [11, 12], and strawberry [13, 14]. Ornamental species (rose, flowering cherry, hawthorn, etc.) are also grown for gardening and residential landscape purposes. In particular, roses in genus *Rosa* and cherry blossom trees in genus *Prunus* are popular plants worldwide for their beautiful blossoms and superior ornamental characteristics. Genus *Prunus* is a member of tribe Amygdaleae that develops a drupe. Despite the large number of *Prunus* species (approximately 250), the majority of flowering cherry species are originally native to eastern Asia, including Korea, Japan, and China, where various natural and artificial hybrids have been developed and selected [15, 16]. Due to a long history of cultivation along with naturalization of wild flowering cherry species and inter-specific hybridization in eastern Asia, there has been confusion over name, origin, and delimitation between taxa.

One of the controversial issues raised in flowering *Prunus* species is the relationship between cultivated and wild taxa of *P. yedoensis* and their relatives. Among the diverse flowering *Prunus* species, the “Yoshino cherry” tree (*P.* × *yedoensis*, Pxy) from Japan is one of the most popular hybrid species that has been extensively planted not only in Japan, but also in many other locations, for example, the Tidal Basin in Washington, DC, USA. The “Yoshino cherry” was derived from a cross between paternal *P. speciosa* and maternal *P. pendula* f. *ascendens* (Ppa) [17]. Meanwhile, wild *P. yedoensis* (*P. yedoensis* var. *nudiflora* Koehne [Pyn]), which is referred to as the wild “King cherry” and has superior flower, cherry, and shape ornamental characteristics, is endemic to Jeju Island, Korea. It was first discovered in 1908 in the natural habitat of Mt. Halla on Jeju Island [18]; several natural populations have been conserved as a National Monument of Korea. Due to its scientific name, there has been confusion about whether the wild “King cherry” and the “Yoshino cherry” are the same taxa or if the “Yoshino cherry” originated from wild *P. yedoensis*. Phylogenetic studies using a limited number of DNA markers suggested that Pyn is closely related to the “Yoshino cherry,” but that these two taxa are distinguishable [19, 20]. However, a genome-level comparison between the two taxa has not been reported so far.

*Prunus* species have a gametophytic self-incompatibility (GSI) system to avoid self-fertilization. The interaction between pollen and the style or ovule of *Prunus* species is determined by a specific pair of S-locus genes, a male determinant S haplotype-specific F-box protein (SFB) and a female determinant S-locus ribonuclease (S-RNase). The two loci are completely linked to maintain the co-evolved allele specificities for the compatibility of male and female gametes; therefore, the *S-RNase* and *SFB* gene pair is referred to as the S haplotype in *Prunus* [21, 22]. Similar to the case of Pxy, Pyn is also likely to be a natural hybrid derived from maternal Ppa and an unknown paternal *Prunus* species [23], suggesting compatibility of the S haplotype between distinct *Prunus* species. Recently, we investigated the genetic structure of the Pyn population along with candidate parental *Prunus* species on Jeju Island using 20 nuclear gene-based DNA markers and found that Pyn may originate from a cross between maternal Ppa and paternal *P. jamasakura* (Pj). In addition, approximately 81% of the wild Pyn accessions examined were likely F1 hybrids, whereas the remaining 19% were backcross hybrids resulting from additional asymmetric introgression of parental genotypes, suggesting that Pyn on Jeju Island is a homoploid inter-specific hybrid [24]. However, there are still additional questions that need to be answered, including the organization of the Pyn genome as a homoploid hybrid and how the putative parental genomes hybridized into the Pyn genome. These concerns can be addressed by a comprehensive and in-depth analysis of the Pyn genome.

With the aim of describing the hybrid genome, which is fundamental for understanding the structure and organization of the hybrid flowering *Prunus* genome, in this study, we report the draft genome assembly, annotation, and analysis of the heterozygous wild Pyn genome based on PacBio RSII long-read sequencing refined with Illumina short-read sequences. We assembled the Pyn genome into “haplotype-fused” long contigs then inferred haplotypes of genic regions by mapping to the short-read sequences of putative parental species. We also conducted whole-genome resequencing analysis of five Pyn accessions and 11 accessions of related *Prunus* species to verify the parental origin and genomic delimitation of hybrid taxa. Comprehensive analysis of heterozygous genome assembly and variation data between genotypes collectively provides novel insights into the organization and hybridization of the wild flowering *Prunus* genomes.

## Results

### Highly heterozygous genomic nature of wild *P. yedoensis*

**Fig. 1:**
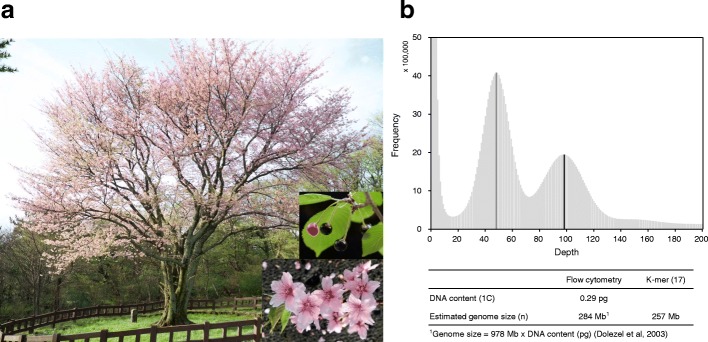
The reference accession of wild *Prunus yedoensis* used in this study. **a** *Photographs* of a Pyn-Jeju2 tree and its flowers and berries taken from March to April 2017. **b** Estimation of the genome size of Pyn-Jeju2 based on K-mer analysis. The *top panel* represents the volume of K-17mer (*Y-axis*) plotted against the frequency at which it occurs (*X-axis*). The *gray* and *black* peaks correspond to heterozygous and homozygous reads, respectively. The *bottom panel* shows the estimated haploid genome size based on the homozygous K-mer peak as well as flow cytometry analysis

Pyn*-*Jeju2 is an endemic wild flowering cherry tree preserved in a natural habitat on Jeju Island, Korea with a beautiful shape, pink flowers, and black berries (Fig. 1a). The nuclear genome of Pyn is organized into eight chromosomes (2n = 2х = 16); the size and structure of Pyn chromosomes are highly similar to those of Ppa and *P. jamasakura* var. *jamasakura* (Pjj) [25]. The genomic characteristics of Pyn*-*Jeju2 were viewed through substring of length K (K-mer) analysis using Illumina short reads at a K-mer size of 17. As shown in Fig. 1b, the frequency distribution of K-mer showed two clear peaks located at coverage 48× and coverage 98×, corresponding to the heterozygous and homozygous reads, respectively. The heterozygous read peak showed a frequency approximately twofold that of the homozygous read peak, demonstrating that Pyn*-*Jeju2 has a hybrid genome. The maximum haploid genome size of Pyn was estimated to be 257 megabases (Mb) based on homozygous reads. Moreover, the heterozygous fraction of the Pyn genome predicted that the maximum diploid genome size of Pyn is 525 Mb. A flow cytometry assay also estimated a similar haploid genome size of 284 Mb (1C = 0.29), which was in accordance with the range of diploid *Prunus* genomes (mean 1C = 0.28) reported in the Plant DNA C-value database [26]. Additional K-mer analysis of four Pyn accessions and four “Yoshino cherry” accessions showed fundamentally similar patterns of K-mer frequency distribution (Additional file 1: Figure S1). These findings collectively suggest that Pyn is a natural homoploid hybrid.

### De novo genome assembly of wild *P. yedoensis*

The main challenge of the Pyn genome for de novo assembly is its high heterozygosity. Considering the hybrid genome structure of Pyn, we applied PacBio RSII sequencing and the FALCON assembler as a long-read sequencing and overlap-layout-consensus assembly strategy (Additional file 1: Figure S2). We generated approximately 18.8 gigabases (Gb) of PacBio RSII reads affording 73-fold coverage of the haploid genome. In addition, 306.6 Gb of Illumina short-read sequences were also generated for correction of the PacBio reads, additional assembly, and scaffolding (Additional file 2: Table S1). After read correction and quality trim, filtered sequences (85.0 Gb) were de novo assembled into 4292 contigs (N50 of 132.6 kilobases [kb]), as well as a complete chloroplast genome that was identical to the previously reported sequence (GenBank accession NC_026980). The initial assembly included both “haplotype-fused” contigs and associate contigs representing highly divergent regions between the homologous sequences. Additional assembly with Illumina mate-paired (MP) reads and Fosmid-end sequences allowed for the generation of the Pyn draft genome assembly, which consisted of 3185 scaffolds (Table 1). The Pyn draft genome assembly was 323.8 Mb (scaffold N50 of 199.0 kb), which was 126.0% of the estimated haploid genome size. Comparing this assembly to transcriptome data revealed that the draft assembly can recover > 93% of the gene space (Additional file 3: Table S2; Additional file 4: Table S3). Analysis of variation based on single nucleotide polymorphisms (SNPs) identified by mapping of Illumina short reads on to the assembly showed that 2.5 Mb (1.1%) of the total contig length was classified as heterozygous.

**Table 1:** Summary statistics of the draft genome assembly of wild *P. yedoensis*

	Contig		Scaffold	
	Length (bp)	Number	Length (bp)	Number
N90	38,284	2435	54,586	1700
N80	59,290	1770	88,524	1239
N70	81,939	1312	124,582	934
N60	106,886	973	158,837	702
N50	132,585	706	198,954	519
Longest	773,088		960,226	
Overall (> 1 kb)	318,739,121	4292	323,781,369	3185

### General features of the wild *P. yedoensis* genome

The statistics of repetitive sequences and protein-coding genes in the Pyn genome are depicted in Table 2, Additional file 5: Table S4, Additional file 6: Table S5, and Additional file 7: Table S6. Sequence analysis of the draft genome assembly showed that the overall proportion of repetitive sequences as well as the number of protein-coding genes were higher in Pyn and sweet cherry (*P. avium*, Pa) than in Chinese plum (*P. mume*, Pm) and peach (*P. persica*, Pp) primarily due to an excessive amount of assembled sequence. Approximately 47.2% of the assembled Pyn genome consisted of repetitive sequences, with 24.9% retrotransposons and 14.9% DNA transposons. Similar to the cases of Pa, Pm, and Pp, the most dominant retrotransposon in the Pyn genome was Ty3/Gypsy followed by Ty1/Copia, whereas CMC-EnSpm was the most abundant DNA transposon (Additional file 5: Table S4). In total, 41,294 protein-coding genes and 2187 RNA genes were predicted from the draft genome of Pyn (Table 2), which is 1.3- to 1.5-fold more genes than the Pm and Pp genomes but slightly fewer genes than the Pa genome. However, the average length of protein-coding genes was shorter than those of the sequenced *Prunus* genomes due to a reduction of exon length. In contrast, the average gene density was 7.7 kb per gene, which is similar to Pm (7.6 kb per gene) but higher than Pp (8.2 kb per gene) and lower than Pa (6.2 kb per gene). Therefore, it is likely that overall genomic organization characteristics of Pyn are similar to those of fruit crop *Prunus* species. Of the predicted genes, 33,802 (81.9%) had at least one match to messenger RNA sequencing (mRNA-seq) reads from the present study. Functional annotation identified 37,444 genes as “known” based on expression, database matches, or any detectable domain signatures, whereas the remaining 3850 (9.3%) were assigned as “unknown” or “hypothetical” (Additional file 7: Table S6). Expression analysis of protein-coding genes revealed that a total of 4287 genes were alternatively spliced in different tissues and that 230 isoforms were tissue-specific alternative splicing variants. The most abundant types of alternative splicing events were alternative transcription start or termination sites (Additional file 8: Table S7).

**Table 2:** Comparison of repetitive sequences and annotated protein-coding genes in the draft assemblies of four *Prunus* genomes

Genome	Characteristics	*P. yedoensis* var. *nudiflora*	*P. avium*	*P. mume*	*P. persica*
Draft sequences	Size (Mb)	318.7	272.4	237.2	227.2
Repetitive sequences	No. RNA genes^a^	2187	729	1541	1243
	DNA TE (Mb)	47.6	26.5	25.5	38.7
	RNA TE (Mb)	79.5	56.4	49.9	62.6
	Simple repeats (Mb)	5.9	5.3	4.2	4.1
	Other repeats (Mb)	5.6	3.9	3.3	2.7
	Total non-redundant bases (Mb)	150.8	103.9	71.1	88.0
Protein-coding genes	Total number	41,294	43,673	31,390	27,864
	Avr. gene size (bp)	2154	2294	2514	2607
	No. exons per gene	4.3	3.6	4.6	5.1
	Avr. exon size (bp)	220	248	249	243
	Avr. intron size (bp)	362	417	380	317
	Avr. gene density (kb/gene)	7.7	6.2	7.6	8.2

Statistics for *P. avium*, *P. mume*, and *P. persica* are based on Shirasawa et al. [8], Zhang et al. [9], and The International Peach Genome Initiative [10], respectively, and repetitive sequences were recalculated using the same criterion used to annotate the *P. yedoensis* var. *nudiflora* genome

^a^Sequences encoding ribosomal RNA, transfer RNA, and microRNA were considered

**Fig. 2:**
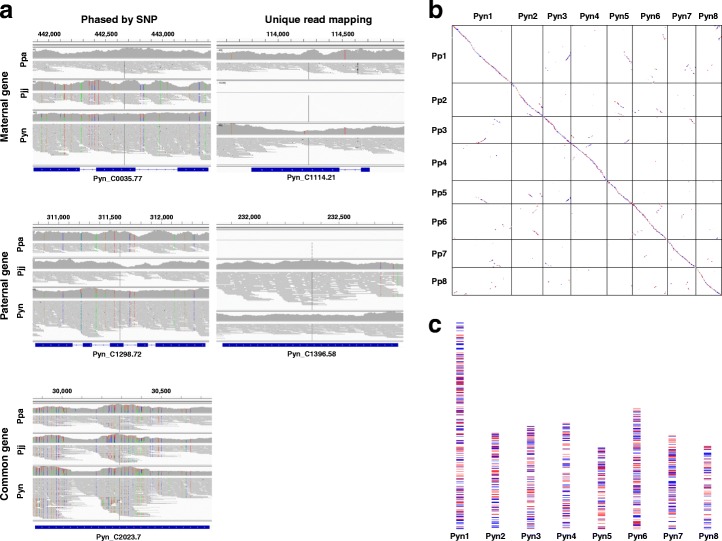
Phasing and arrangement of the heterozygotic genome assembly. **a** Examples of haplotype-phased gene models. Gene models predicted from the initial “haplotype-fused” assembly are phased according to read mapping and SNP analysis using the Illumina short-read sequences of putative parental species. Genes were phased into one parental haplotype if a gene was aligned only by reads from one parental species (unique mapping) or had at least twofold as many supports for SNPs by reads of one parental species (phased by SNP). Genes with similar supports of read mapping for both parental species are defined as common type. *Colored dots* denote SNPs identified in the aligned reads. **b** Chromosomal arrangement of the gene-phased genome assembly of wild *P. yedoensis* (Pyn) onto the *P. persica* (Pp) genome. **c** Distribution of haplotype-phased genes in the tentative chromosomes of wild *P. yedoensis*. *Colored dots* or *lines* represent maternal-phased genes (*red*), paternal-phased genes (*blue*), or common genes (*gray*)

Since the draft Pyn genome was assembled into “haplotype-fused” scaffolds as well as variant sequences that exceed the estimated haploid genome size, we could phase the gene models by mapping the short-read sequences of candidate parental species, Ppa (Ppa-1) and Pjj (Pjj-1), even though the FALCON Unzip algorithm was unavailable when the draft genome was assembled (Fig. 2a). Phasing of genes based on SNP analysis with parental sequences showed that approximately 59.2% of genes were encoded in the homozygous sequence fraction and the remaining 40.8% of total genes were phased into either maternal (19.4%) or paternal (21.4%) origin (Table 3). Although Pyn*-*Jeju2 is a wild accession and none of its genetic resources, including a genetic map, were available, we tentatively assigned and ordered the scaffolds to the chromosome sequences of Pp as reference using BLAST and MCScanX [27] comparisons. The chromosome-assigned Pyn sequences covered 281.6 Mb, which included 87.0% of the draft assembly, and showed one-to-one chromosome level syntenic matches with Pp (Fig. 2b), Pa, and Pm genomes (Additional file 1: Figure S3). The chromosome-assigned sequences of Pyn covered 54.5%–66.4% of the sequenced *Prunus* genomes (Additional file 9: Table S8). The distribution of phased genes along the tentative Pyn chromosomes demonstrated a complicated mosaic pattern (Fig. 2c). These findings indicate that Pyn has a hybrid genome derived from Ppa and Pjj where parental sequences are organized into a hybrid genome with a random arrangement.

**Table 3:** Classification of wild *P. yedoensis* genes based on sequence phasing of the draft assembly by mapping of Illumina short-read sequences from putative parental species, maternal *P. pendula* f. *ascendens*, and paternal *P. jamasakura*

Type	Maternal gene		Paternal gene		Common gene
	Unique	Phased	Unique	Phased	
Number	548	7482	1353	7456	24,455
Ratio (%)	1.3	18.1	3.3	18.1	59.2

### Comparative genome analysis of wild *P. yedoensis*

**Fig. 3:**
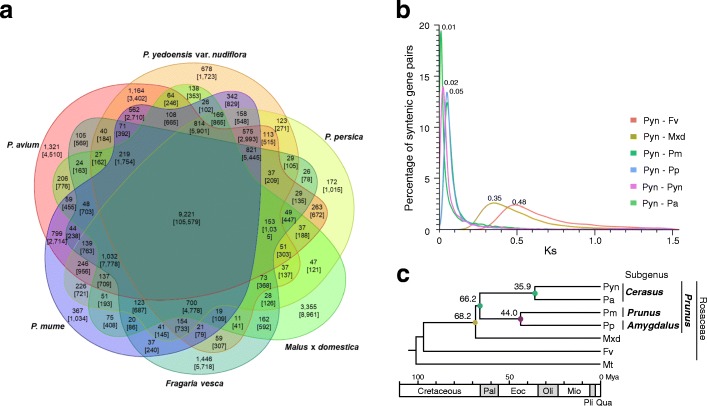
Comparative analysis of the wild *P. yedoensis* var. *nudiflora* genome. **a** *Venn diagram* showing the unique and shared gene families between six sequenced genomes of the Rosaceae family. The number of gene families and genes (in bracket) for each group are shown. **b** Distribution of Ks values obtained from comparisons of orthologous gene sets between six genomes of the Rosaceae family and paralogous gene sets in wild *P. yedoensis*. **c** Genome evolution of *Prunus* species. Estimation of dates for speciation events are given in millions of years based on Bayesian evolutionary analysis of 276 conserved single copy genes. Pyn *P. yedoensis* var. *nudiflora*, Fv *Fragaria vesca*, Mt *Medicago truncatula*, Mxd *Malus* × *domestica*; Pa *P. avium*, Pm *P. mume*, Pp *P. persica*. Pal Paleocene, Eoc Eocene, Oli Oligocene, Mio Miocene, Pli Pliocene, Qua Quaternary

Comparative analysis of the Pyn genome revealed unique characteristics of the flowering cherry genome. A six-way comparison of Pyn, Pa, Pm, Pp, strawberry (*Fragaria vesca*, Fv), and apple (*Malus* × *domestica*, Mxd) genes using OrthoMCL analysis yielded 16,777 Pyn gene families (30,478 genes), of which 9221 (55.0%) were shared by all six species. However, the analysis revealed 678 gene families (4.0%) consisting of 1723 genes that were unique to Pyn (Fig. 3a). Gene annotation revealed 273 gene families (650 genes) as “known” with the remaining 405 gene families (1073 genes) assigned as “unknown” or “hypothetical.” Comparison of gene families with the fruit crop *Prunus* genomes identified specific over-represented or under-represented (*p* < 0.001) gene families in the Pyn genome. For example, the P-loop-containing nucleoside triphosphate hydrolase superfamily genes and the C3HC zinc finger-like genes were enriched, whereas the glycosyl hydrolase family and the zinc ion binding protein genes were under-represented in the Pyn genome (Additional file 10: Table S9). Of particular interest, NAC transcription factors (TF), auxin response factors, SNF2/Brahma-type chromatin remodeling protein, and plant neutral invertase genes were almost twofold more abundant in the Pyn genome compared to other fruit crop *Prunus* species. In addition, FLOWERING WAGENINGEN and Early Flowering 6 TFs that regulate flowering time were also abundant in the Pyn genome.

Divergence of the Pyn genome from the tribe Potentilleae (Fv), Maleae (Mxd), and fruit crop *Prunus* in subgenus *Amygdalus* (Pp), *Prunus* (Pm), and *Cerasus* (Pa) genomes was deduced based on the synonymous substitution rate (Ks) of homologous genes and Bayesian evolutionary analysis. As shown in Fig. 3b, splitting of the Pyn genome from its close relatives was conducted by comparing orthologous genes. Pyn shared a single peak with Fv and Mxd at Ks modes of 0.48 and 0.35, respectively, indicating successive splitting of the *Prunus* lineage from Potentilleae and Maleae presumably during the Cretaceous and Paleocene periods around 88 to 61 million years ago (Mya) [28]. The Ks distribution between *Prunus* genomes showed a very recent diversification of *Prunus* species. The peaks at a Ks mode of 0.05 for orthologs between Pyn-Pm and Pyn-Pp genomes were essentially identical. Moreover, paralogs of the Pyn genome and orthologs between Pyn-Pa showed a single peak at Ks modes of 0.02 and 0.01, respectively, demonstrating successive splitting of the Pyn genome from subgenera *Amygdalus* (Pp), *Prunus* (Pm), and *Cerasus* (Pa). Bayesian evolutionary analysis of 276 single copy orthologous genes conserved in six Rosaceae species and one outgroup Fabaceae species (*Medicago truncatula*, Mt) identified 376,758 aligned positions with 127,606 (33.7%) variable sites, 29,134 (7.7%) of which were parsimoniously informative. Figure 3c shows a chronogram estimating divergence time. Molecular dating analysis performed with BEAST estimated the age of genus *Prunus* at approximately 66.2 Mya (95% higher posterior densities [HPD] of 64.2–67.4 Mya). Similarly, the age estimate for the split of subgenera *Prunus* and *Amygdalus* was around 44.0 Mya (95% HPD of 42.2–45.6 Mya) and the divergence time between Pyn and Pa was estimated to be around 35.9 Mya (95% HPD of 34.4–37.3 Mya). These results were consistent with a previous report on the rapid diversification of *Prunus* lineage of eastern Asian origin, presumably 35 Mya [28].

### Expression of lineage-specific genes in the inter-specific hybrid genome

**Fig. 4:**
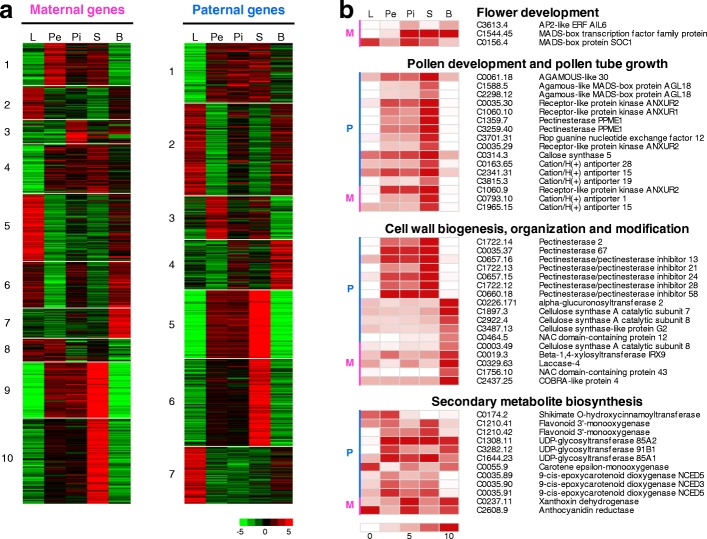
Differential expression of haplotype-phased genes in various tissues of wild *P. yedoensis*. **a** *Heat maps* representing the expression of 562 maternal- and 576 paternal-phased genes, which are identified as differentially expressed genes in the mRNA-seq analysis, in different tissues. The normalized count values of a given gene from three independent biological replicates across all samples were used as a normalization factor. The vertical axes organize genes according to co-expression. The *horizontal axes* represent five tissues: leaf (L), petal (Pe), pistil (Pi), stamen (S), and berry (B). **b** *Heat maps* showing the differential expression of a selected category of genes related to development and secondary metabolite biosynthesis. The average normalized count values represent the relative expression across tissues. M maternal-phased genes, P paternal-phased genes

In hybrid organisms, genes are inherited from the two parents; differences in gene expression or modification may result in hybrid vigor or weakness. To determine the expression characteristics of the genes originating from a single parental lineage, we performed mRNA-seq analysis and expression profiling in five tissues, namely, leaf, petal, pistil, stamen, and berry (Additional file 3: Table S2). Overall, genes inherited from only one parental lineage (86.5%) were more abundantly expressed than those from both parental lineages (78.6%). Moreover, approximately 7.0% (562) of maternal genes and 6.5% (576) of paternal genes were differentially expressed in various tissues (Fig. 4a). Most differentially expressed genes (DEGs), except for two maternal genes and two paternal genes, were expressed in more than two tissues, demonstrating less tissue specificity. Clustering analysis and a functional enrichment study of DEGs for each parental lineage also showed that several gene groups that originated from only one parental lineage were outnumbered and expressed differentially in specific tissues (Fig. 4b). For example, three flower development-related TF (AP2-ERF and MADS-box TFs) genes that originated from the maternal lineage were differentially expressed in vegetative and reproductive tissues; however, no DEGs from the paternal lineage were identified. Meanwhile the paternal lineage had 2.5- to 5-fold more DEGs for groups of genes related to pollen or pollen tubes, the cell wall, and secondary metabolite biosynthesis than those from the maternal lineage. Regardless of the parental origin, these genes were more abundantly expressed in the three floral and berry tissues than in the leaf tissue.

### Resequencing analysis of wild *P. yedoensis* and its related species

To study the genomic relationship between Pyn and its closely related species, we resequenced five Pyn accessions, four “Yoshino cherry” accessions (Pxy), and seven accessions of candidate parental species, including Ppa, Pj (Pjj and *P. jamasakura* var. *quelpaertensis* [Pjq]), and *P. sargentii* (Psa), and compared this resequencing data with the reference assembly of Pyn-Jeju2 (Table 4; Additional file 11: Table S10). The paired-end (PE) reads of Pyn-Jeju2 itself covered 99% of its reference assembly. In addition, at least 91% of PE reads from other examined *Prunus* species were successfully mapped onto the reference genome of Pyn, covering 82–96% of the genome. This excludes Ppa-2 and Pjj-2, for which the genomes were sequenced at low coverage (7.5×). Read mapping rate and reference genome coverage were the highest for Pyn genotypes, followed by Pxy and parental taxa.

**Table 4:** Summary of SNP and InDel variations in *Prunus* species

Taxon	Genome coverage (%)	Read mapped rate (%)	Variation				SNP in CDS					InDel in CDS		SNP + InDel	
			Hetero SNP^a^	Homo SNP^b^	InDel	Overall	Silence	Nonsense	Missense	Splicing site	Ti/Tv	In-frame	Frame shift	Intron	Intergenic
Pyn-Jeju1	95.4	94.4	2,838,985	1,096,549	895,813	4,831,347	148,300	3661	182,107	1158	1.56	4340	22,281	255,057	4,214,443
Pyn-Jeju2	98.6	95.6	2,596,728	145,287	764,810	3,506,825	99,482	2560	123,473	851	1.52	2967	20,894	180,133	3,076,465
Pyn-Jeju3	95.7	93.9	2,946,741	1,262,874	932,447	5,142,062	153,376	3679	187,285	1259	1.57	4421	22,453	269,721	4,499,868
Pyn-Jeju4	95.3	94.5	2,896,646	1,223,511	916,719	5,036,876	152,756	3652	186,991	1222	1.57	4337	22,262	266,970	4,398,686
Pyn-Jeju5	94.5	93.9	3,378,621	1,657,723	1,063,188	6,099,532	176,065	4532	218,722	1501	1.58	5187	23,776	313,087	5,356,662
Pxy-US1	95.0	94.6	3,464,156	1,629,328	1,072,679	6,166,163	175,976	4596	220,018	1527	1.58	5244	23,717	315,788	5,419,297
Pxy-US2	94.9	94.5	3,445,971	1,626,652	1,068,387	6,141,010	175,718	4600	219,181	1490	1.58	5195	23,653	314,928	5,396,245
Pxy-JP1	94.9	91.3	3,527,140	1,722,941	1,109,412	6,359,493	182,173	4620	225,405	1538	1.58	5329	24,706	324,046	5,591,676
Pxy-JP2	94.9	92.6	3,529,437	1,724,190	1,110,969	6,364,596	182,189	4639	225,537	1557	1.58	5343	24,780	324,679	5,595,872
Ppa-1	82.2	93.3	1,197,274	1,428,540	595,858	3,221,672	87,944	2581	116,320	819	1.60	2857	14,995	165,140	2,831,016
Ppa-2	72.0	94.0	726,505	1,263,932	482,136	2,472,573	74,707	1966	94,308	660	1.56	2216	13,013	136,354	2,149,349
Ppa-3	84.6	96.0	1,883,696	1,522,293	751,829	4,157,818	118,186	3088	148,056	1001	1.59	3340	17,363	218,562	3,648,222
Pjj-1	86.9	93.8	1,975,049	1,823,000	776,618	4,574,667	144,854	3306	173,239	1120	1.55	4011	17,776	238,548	3,991,813
Pjj-2	76.1	94.8	1,139,292	1,592,058	583,439	3,314,789	120,474	2333	138,885	837	1.52	3104	14,767	183,366	2,851,023
Pjq	86.4	94.0	1,946,474	1,769,052	763,036	4,478,562	145,116	3211	172,508	1119	1.55	3924	17,563	233,810	3,901,311
Psa	86.6	93.7	1,960,926	1,823,069	775,824	4,559,819	144,689	3312	173,070	1131	1.55	3957	17,591	240,595	3,975,474
Total	–	–	39,453,641	23,310,999	13,663,164	76,427,804	2,282,005	56,336	2,805,105	18,790	–	65,772	321,590	3,980,784	66,897,422

^a^Heterozygous SNP rate, proportion of heterozygous SNPs in a genome

^b^Homozygous SNP rate, proportion of homozygous SNPs in a genome

Ti transition, Tv transversion

**Fig. 5:**
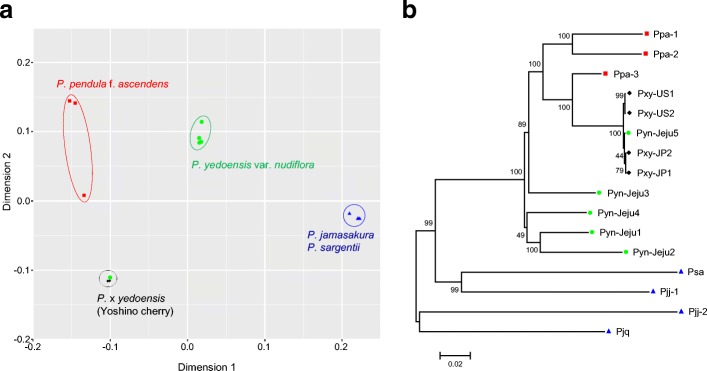
The genomic relationships between flowering *Prunus* taxa. **a** Multidimensional scaling of *Prunus* accessions. Closely related accessions of Ppa (*red square symbol*), Pyn (*green circle symbol*), Pj or Psa (*blue triangle symbol*), and Pxy (*black diamond symbol*) are grouped together using *dotted circles*. **b** A maximum likelihood tree of *Prunus* accessions based on SNPs/InDels identified by variome analysis. The accession names are presented in Additional file 11: Table S10

In total, 76,427,804 SNPs and insertions or deletions (InDels) were identified by multi-sample variome analysis of all 16 resequencing samples (Table 4). Since all the accessions were wild accessions with heterozygous genomes (Additional file 1: Figure S1), the heterozygous SNP rate was approximately 52% of the total variome. Pxy accessions showed the most diverse genotypes with 6.3 million SNPs/InDels on average. In contrast, parental accessions had less variation (3.3 million variations for maternal and 4.2 million variations for paternal taxa on average) than Pyn accessions (4.9 million variations on average). There was no significant difference in variome size between the paternal taxa, Pjj, Pjq, and Psa. The transition/transversion (Ti/Tv) ratio in *Prunus* species was 1.52–1.60. Interestingly, the Pyn-Jeju5 accession had 6.1 million variations, which was a similar level to Pxy accessions. Multidimensional scaling (MDS) and a maximum likelihood (ML) tree based on variome data indicated that the Pyn accessions were distinctly separated from the Pxy accessions, except for Pyn-Jeju5 (Fig. 5). The Pyn-Jeju5 accession was grouped together with the Pxy accessions on both the MDS plot and the ML tree, showing that this accession is more closely related to Pxy. Of particular interest, the Pyn accessions were located in the middle of maternal Ppa accessions and paternal Pj and Psa accessions by both dimensions of MDS, demonstrating that the Pyn accessions have intermediate genomic characteristics of the two parental groups.

### Haplotype analysis of S-locus genes in flowering *Prunus* taxa

**Fig. 6:**
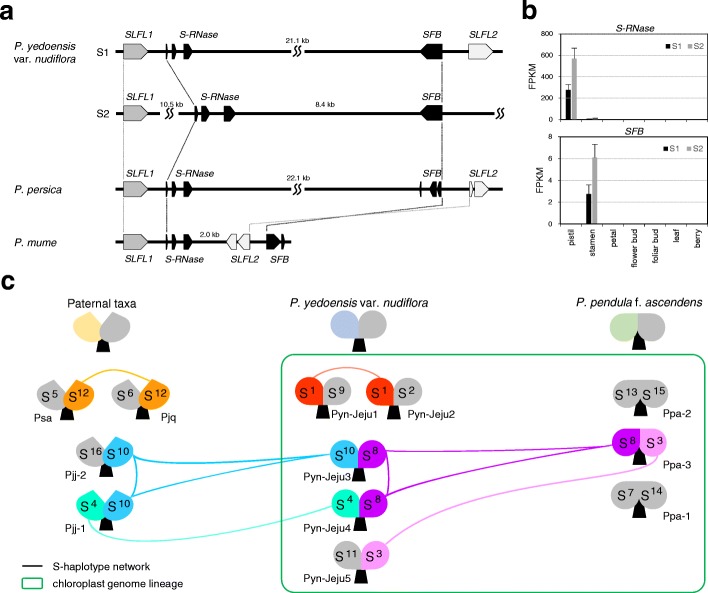
Characterization of S haplotypes in flowering *Prunus* species. **a** Microsynteny at the S-locus regions between wild *P. yedoensis* and fruit crop *Prunus* (*P. persica* and *P. mume*). There are two S haplotypes in the heterozygous Pyn genome (31 kb of S1 and 35 kb of S2) compared to a single S haplotype in the homozygous Pp and Pm genomes. Syntenic genes are connected with lines. **b** Relative expression levels of the *S-RNase* and *SFB* genes in different tissues are presented by the average fragments per kilobase million (FPKM) value from three independent biological replicates. **c** S haplotype network in a natural *Prunus* population. A total of 15 S haplotypes from 12 accessions, which are distributed sympatrically in a natural habitat on Jeju Island, were identified. Accessions are placed according to their relative geographic location in the natural habitat. Shared S haplotypes between accessions are connected with *lines* of the same color. Chloroplast genome lineage, showing < 10 nucleotide differences in the protein-coding sequences of the whole chloroplast DNA (Additional file 12: Table S11), is also presented in the *green box*

Considering the hybrid genome structure of Pyn, we performed comparative analysis of the S-locus between accessions of Pyn and the possible parental species distributed within approximately 3 km in a natural forest on Jeju Island (Fig. 6). The reference genome assembly of Pyn-Jeju2 included two S-locus haplotypes, named S1 and S2. The gene structure of the S1 haplotype was syntenic to that of Pp, consisting of *S-RNase* and *SFB* genes flanked by *S-locus F box-like1* (*SLFL1*) and *SLFL2*. The S2 haplotype also showed the same order of genes except for *SLFL2*, which was not predicted across the 11-kb downstream region (Fig. 6a). Expression of S-locus genes was characterized by tissue specificity. *S-RNase* and *SFB* genes were expressed only in pistil and stamen, respectively, with approximately twofold expression of S2 haplotype genes (Fig. 6b).

Using Illumina reads of wild *Prunus* accessions, we assembled the S-locus regions of four Pyn, three Ppa, two Pjj, one Pjq, and one Psa accessions and compared them with those of the Pyn*-*Jeju2 reference genome. All the S-locus haplotypes of each accession were heterozygous with pairs of *S-RNase* and *SFB* genes, showing obligate out-crossing. Sequence comparison and phylogenetic analysis of *S-RNase* and *SFB* genes in the S-locus regions classified 15 S haplotypes (Fig. 6c; Additional file 1: Figure S4). None of the accessions had the same combination of S haplotypes; however, six S haplotypes (S1, S3, S4, S8, S10, and S12) were shared among ten accessions. Of particular interest, each S haplotype from two Pyn accessions (Pyn-Jeju3 and Pyn-Jeju4) was directly linked to its counterpart in the genomes of one maternal (Ppa-3) and two paternal (Pjj-1 and Pjj-3) accessions. In contrast, paternal taxa shared none of the S haplotypes with maternal accessions. Comparison of the chloroplast genomes of the accessions supported the relationship between Pyn and Ppa in the maternal lineage (Additional file 12: Table S11). Overall, these findings suggest that the compatibility of S-locus haplotypes of maternal Ppa and paternal Pj could result in generation of Pyn as an inter-specific homoploid hybrid.

## Discussion

In the research on plant genomes, high-quality reference genome assembly serves as a fundamental resource for various genomic studies. However, most assemblies of heterozygous genomes, such as out-bred, wild-type hybrid, and polyploid organisms, generated by short-read sequencing tend to produce a more fragmented assembly compared to homozygous genomes of similar size or complexity. Consequently, the utility and applications of heterozygous genomes with fragmented assemblies have been limited [29]. Approaches to developing an adequate solution for the sequencing and assembly of heterozygous genomes include sequencing both parents and offspring to infer haplotypes [30] and long-read sequencing combined with a phased diploid genome assembler [5].

A number of *Prunus* species have been identified as natural or artificial hybrids, many of which are fruit, nut, or ornamental crop species, providing genetic diversity in the Rosaceae family [15]. Hybrid *Prunus* species have been characterized either by phenotypic or genetic characteristics; however, no hybrid *Prunus* genomes have been characterized to date. In this study, we sequenced and analyzed the genome of Pyn, a hybrid flowering cherry, and provided sufficient information to solve the controversial issues surrounding this taxon, including parental origin, degree of hybridity, and taxon boundary against related species. Furthermore, this is the first report to provide valuable reference sequences for flowering cherry plants in the genus *Prunus* of the Rosaceae family for plant genomics and evolutionary analyses. More importantly, we successfully de novo assembled the heterozygous genome of a wild hybrid taxon. For the genome sequencing and assembly of Pyn, we used a long-read sequencing and assembly strategy combined with haplotype phasing using short-read sequences of putative parental species, Ppa and Pjj. The initial assembly was constructed using a FALCON assembler as “haplotype-fused” contigs and their associated heterozygous structural variants.

The overall assembly quality of the heterozygous genome was sufficiently high for the downstream analysis, including genome comparison, variome analysis, and expression profiling, on the basis of 4.2- and 481.9-fold longer N50 length of contiguous assembled sequences (133 kb) than that of the Pm (32 kb) and Pa (276 bp) genomes, respectively, which were assembled as a homozygous haploid genome using Illumina short-read sequences [8, 9]. Genome level correspondence of the Pyn assembly to the eight chromosome pseudomolecules of Pa, Pm, and Pp also revealed the quality of the Pyn assembly. We anticipate that long-read sequencing and assembly is sufficiently effective to sequence, assemble, and analyze highly heterozygous wild plant genomes. Meanwhile, the assembly contiguity of Pyn was less than that of Pp (294 kb), which was assembled based on Sanger sequencing data [10], presumably due to the absence of contig ordering and scaffolding by intensive genetic mapping. We included Illumina short-read PE and MP sequences as well as Fosmid-end sequences to order and scaffold the contigs; however, the assembly was not significantly improved. Therefore, we expect that the contiguity of long-read assembly for wild plants can be improved through the use of genome-wide chromatin interaction data such as Hi-C [31].

It is noteworthy that the haplotype phasing of an assembly using putative parental species enabled us to investigate haplotype structures and the hybridity of Pyn. Phasing of genic regions of Pyn showed that approximately 41% of gene models were phased into maternal Ppa (19.4%) and paternal Pjj (21.4%) haplotypes. We further demonstrated that the Pyn accessions were grouped together with a similar distance to the Ppa and Pj groups by two dimensions in the MDS plot. Together with the almost identical chloroplast sequences of Pyn and Ppa accessions, these findings provide strong evidence supporting our previous suggestion that Pyn is likely an F1 hybrid taxon resulting from a maternal Ppa and paternal Pj cross. Of particular interest, Psa was clustered with Pjj and Pjq in both the MDS plot and the ML tree, suggesting that Pyn may have an additional source of paternal lineage compared to the single maternal lineage from Ppa. Additionally, the haplotype-specific genes showed abundant expression in various Pyn tissues. We assume that these lineage-specific genes, together with common genes originating from both parental lineages, may exert a synergistic effect to produce the superior ornamental characteristics of Pyn as a natural hybrid.

With respect to delimitation of the taxon boundary, genome-wide variome analysis using the Pyn assembly as a reference also provided a precise clue to the genetic relationship between Pyn and Pxy. Whole-genome resequencing, MDS plot, and ML tree analyses revealed that Pyn accessions were clearly separated from Pxy accessions. Pxy showed significant genome-level variation from Pyn and Pj accessions but showed a relatively close relationship to Ppa. This finding strongly suggested that Pyn and Pxy have a distinct paternal background. Interestingly, one accession of Pyn (Pyn-Jeju5) was tightly grouped with Pxy in both the MDS plot and the ML tree. Although this accession grows in a natural forest on Jeju Island, we assume that Pyn-Jeju5 is an accession of Pxy that escaped from the cultivated area. Considering the fact that Pyn-Jeju5 has been taxonomically classified as Pyn based on morphological characters, highly precise tools such as molecular markers should be utilized to correctly identify Pyn for evaluation and conservation of this taxon in nature.

For reproduction of *Prunus* species, successful pollination, fertilization, and seed formation are indispensable since parthenocarpic fruits have not developed in *Prunus* species. Our comparative genome analysis indicated that diversification of the *Prunus* genomes arose during the Paleocene (up to 66 Mya), followed by the successive split of *Prunus* species during the Eocene (36–44 Mya). This result is supported by the discovery of fossilized endocarps of *P. wutuensis* in eastern Asia dating to the Eocene [32]. Expansion of a specific category of genes related to auxin response and early flowering may have been involved in the diversification of flowering *Prunus* species. In addition, characterization of S-locus haplotypes, the determinant of *Prunus* GSI, in Pyn and its related species has yielded novel insights into the inter-specific hybridization of flowering *Prunus* in sympatric regions. All the flowering *Prunus* accessions investigated in this study had unique pairs of heterozygous S haplotypes that suggested out-breeding. Nevertheless, two Pyn accessions retained a combination of parental S haplotypes, each of which was shared with one parental lineage, and several S haplotypes were also shared between accessions or even different taxa (Psa and Pjq). Considering the fact that these taxa are sympatrically distributed in a 3 km range on Jeju Island, and that their blooming time (late-March to mid-April) and flowering periods (approximately two weeks) overlap, we anticipate that cross pollination between closely related taxa resulted in a hybridization network of flowering *Prunus* in this natural habitat. These findings are consistent with our previous hypothesis that inter-specific hybridization and additional introgression by backcross between closely related flowering *Prunus* species on Jeju Island may produce a hybrid swarm [24]. Therefore, we anticipate that reproductive barriers between closely related flowering *Prunus* genomes are likely to be unestablished, presumably due to strong GSI, resulting in reduced restriction of inter-specific hybridization.

## Conclusions

Hybridization has greatly increased plant diversity by generating new genetic combinations and genomes. However, the highly heterozygous genomic nature of hybrid species complicates genome studies due to the presence of highly similar sequences, a significant challenge in plant genomics. In this study, we successfully sequenced and assembled the draft genome of *P. yedoensis* var. *nudiflora*, a wild hybrid taxon of flowering *Prunus*, using long-read sequencing and a sequence phasing strategy. The results suggest that inter-specific hybridization due to the strong gametophytic self-incompatibility of flowering *Prunus* species may have contributed to the establishment of a natural hybrid taxon. The genome assembly of this taxon, along with the whole-genome resequencing data of related *Prunus* taxa, will provide valuable genomic resources for research, conservation, and breeding studies of *Prunus* species, benefiting both basic and applied plant biologists.

## Methods

### Plant material and genome sequencing

The No. 2 accession at the natural habitat of *P. yedoensis* var. *nudiflora* in Bongae-dong, Jeju Island (the Korea National Monument No. 159, Pyn*-*Jeju2; Fig. 1a) was chosen for genome sequencing. It was estimated to be 200 years old. Genomic DNA (gDNA) was extracted from young leaves collected in April 2015 using a DNeasy Plant Maxi Kit (Qiagen, Valencia, CA, USA) and then used for short-read sequencing. Separately, high molecular weight DNA was isolated according to the nuclei isolation method for single-molecule sequencing [33]. In total, 325.3 Gb of sequence data was obtained using Illumina and PacBio platforms (Additional file 2: Table S1) according to the manufacturer’s protocols. For short-read sequencing, 205.5 Gb of Illumina sequences was generated using NextSeq and MiSeq (Illumina, San Diego, CA, USA) sequencing of 250-base pair (bp) (NextSeq) and 500-bp (MiSeq) insert libraries for PE sequencing and 3-, 5-, 10-, 15-, and 20-kb insert libraries for MP sequencing. In addition, one Fosmid library consisting of 55,200 clones with an average insert size of 40 kb (8.6×) was constructed using the NxSeq 40 kb Mate pair cloning kit (Lucigen, Middleton, WI, USA). Fosmid-end sequences were generated using HiSeq X Ten (Illumina, San Diego, CA, USA). Illumina reads from each library were collected at a minimum quality score (Q20) and then filtered for adaptor contamination and low-quality regions using Trimmomatic v0.32 software [34]. Polymerase chain reaction (PCR) duplicates were removed by FastUniq v1.1 software [35] with a default parameter. Adaptor sequences and PCR duplicates of MP reads were filtered using NextClip software [36]. For single-molecule sequencing, 18.8 Gb of PacBio sequences was generated from a 20-kb library using a PacBio RSII sequencer (Pacific Biosciences, Menlo Park, CA, USA) and sequence errors were corrected using the PBcR pipeline from Celera Assembler 8.3.1 software [37] with default parameters. All of the sequence data generated in this study are summarized in Additional file 2: Table S1.

### Genome size estimation and de novo assembly

The genome size of Pyn was estimated both by flow cytometry and K-mer analysis. For flow cytometry analysis, genome size was measured according to previous reports [38, 39] using a CyFlow Space system (Partec BmbH, Münster, Germany) and diploid *Raphanus sativus* cv. WK10039 (1C = 0.6 pg) as a reference. For K-mer analysis, the occurrences of K-mer with a peak depth were counted using Illumina PE reads, and genome size was calculated by dividing the total read length by coverage of K-mer peak using JELLYFISH 2.1.3 software [40] with K-mer 17. To assemble the sequence reads into a draft genome, a hierarchical hybrid assembly strategy was used (Additional file 1: Figure S2). First, PacBio reads were assembled into the initial scaffolds using the FALCON assembler v0.3.0 with parameters of length cutoff 12 kb, max difference 100, max coverage 150, and minimum coverage 2. The initial scaffolds were filtered with chloroplast and mitochondrial sequences using NUCmer in the MUMmer 3 package [41]. Next, Illumina reads were assembled with the chloroplast and mitochondria-filtered scaffolds to extend the scaffolds using SOAPdenovo2.04 software [42]. The resulting scaffolds were aligned with PacBio reads that had not been assembled into the initial scaffolds for additional assembly and gap filling using PBJelly 15.2.20 software [43] with minMatch 8, minimum identity 70, maxScore 500, noSplitSubreads, and support stage–capturedOnly parameters. Finally, draft assembly was improved by Pilon software [44] for additional gap filling and sequence error correction with fix bases, gaps, and diploid parameters.

### Genome annotation

For gene prediction, a combination of ab initio and evidence-based gene predictions was used. The genome assembly was premasked first for class I and class II transposons using RepeatMasker 4.0.5 [45], RepeatModeler 1.0.8 [46], and LTR_FINDER v1.05 software [47], then protein-coding genes were predicted ab initio using BRAKER1 v1.8 [48], GlimmerHMM v3.0.2 [49], and SNAP [50] programs with parameters trained using the *A. thaliana* matrix. Genes with < 300 bp of coding sequence or an incomplete coding region were filtered out. Predicted proteins with a top match to transposon-encoded proteins [51] and putative gene splits predicted on the unfinished gaps were also excluded from the annotation and gene counts. For evidence-based gene prediction, seven tissues, including floral bud, foliar bud, leaf, petal, stamen, pistil, and berry, were used to generate approximately 223.7 million filtered Illumina PE mRNA-seq reads (Additional file 3: Table S2). Total RNA was isolated from each tissue using the cetyl trimethylammonium bromide (CTAB) method [52], which was then used for mRNA purification, construction of a library with a 500-bp insert size, and sequencing according to the manufacturer’s instructions. Quality filtering of mRNA-seq reads was performed according to our previous study [53]. The resulting transcript sequence data were aligned to the genome assembly and evidence-based gene sets were predicted using the PASA package [54]. We also aligned the gene models of Chinese plum (Pm) [9], peach (Pp) [10], strawberry (Fv) [13], apple (Mxd) [7], and *A. thaliana* (TAIR10) [55] to the genome assembly using Exonerate 2.2.0 software [56]. EVidenceModeler software [54] was used to combine ab initio gene models, transcript alignments, and coding sequence alignments into consensus gene model sets. RNA genes were identified using Infernal [57] for tRNAs, BLASTN search for rRNAs, and sequence comparison using miRBase [58] for miRNAs. The predicted protein-coding genes were annotated based on SwissProt and TrEMBL databases from UniProt [59], RefSeq Plant, and nucleotide databases of the National Center for Biotechnology Information (NCBI) [60] using BLASTP with an E value cutoff of 1E^−10^ and query coverage of 70%. The InterPro database [61] was also used to annotate motifs and domains and the gene ontology information for each gene model was extracted from InterPro.

### Transcriptome and comparative genome analysis

Expression analysis of gene models in five tissues (leaf, petal, stamen, pistil, and berry) was performed using the mRNA-seq reads. PE reads were end-to-end aligned to the coding sequences (CDSs) of gene models using STAR 2.5.2b software [62] with default parameters. Reads that were mapped to multiple locations were excluded. The resulting mapped reads for each gene were normalized and patterns of gene expression between tissues were analyzed using DESeq2 [63] and MCLUST version 3 [64] that are included in the R/Bioconductor package. The data of three biological replicates were pooled and the average normalized read count values for genes were extracted and analyzed. Alternative splicing variants were analyzed using Cuffdiff2 [65] and spliceR [66]. A genome-wide synteny comparison between Pyn and Pa, Pm, or Pp was performed based on an all-against-all BLASTP comparison (E value cutoff of 1E^−10^) and synteny regions were inferred using the MCScanX toolkit [27] with a match score of 50, match size of 5, and gap penalty of − 1. Orthologous gene families among the *Prunus* genomes were identified using OrthoMCL v2.0 software [67] with all-against-all BLASTP (E value cutoff of 1E^−5^ and > 50% match) searches. Gene family comparison between the *Prunus* genomes was performed using the PLAZA 3.0 Dicots database [68]. Genome to genome synteny blocks of Pyn versus Pp, Pa, and Pm were plotted using an in-house perl script. For phylogenetic analysis of gene families, the amino acid sequences of orthologous genes were aligned using MUSCLE v3.8.31 software [69] with default parameters. Aligned sequences were trimmed at both ends using GBLOCKS 0.91b software to eliminate regions of poor alignment [70]. Conserved blocks of aligned sequences were concatenated into a single sequence and phylogenetic trees were constructed using the ML method in MEGA7 software [71]. The stability of each tree node was tested by bootstrap analysis with 1000 replicates. Ks between homologous genes was determined using the PAML package [72].

### Molecular dating

To estimate the divergence time of the *Prunus* genomes, a total of 276 single copy orthologous genes, conserved in four *Prunus* species (Pyn, Pa, Pm, and Pp), two Rosaceae species (Mxd and Fv), and one outgroup Fabaceae species (Mt), were selected using reciprocal BLASTP with an E value cutoff of 1E^−10^ and query coverage of 70%. For Bayesian evolutionary analysis, BEAST v1.7 [73] was used. Input files were generated by BEAUti interface with a GTR + I + G model and the combined dataset was applied for the BEAST analysis with a Yule speciation tree and an uncorrelated lognormal molecular clock model. We constrained the crown age of Rosaceae-Fabaceae with a uniform distribution from 100 to 107 Mya, following Moore et al. [74]. In addition, we applied two calibration points, following Chin et al. [28], each with a uniform distribution as follows: (1) the crown age of Rosaceae at 84.2–92.8 Mya; and (2) the crown age of *Prunus* at 51.6–65.2 Mya. Posterior distributions of parameters were approximated using two independent MCMC analyses of 20,000,000 generations with a 10% burn-in. The results were verified using Tracer v1.5 [75] to ensure that plots from the two analyses converged on the same area and then combined. The samples from the posterior analysis were summarized on a maximum clade credibility tree, which had the maximum sum of posterior probabilities on its internal nodes using TreeAnnotator v1.5.4 [73] with the posterior probability limit set to 0.5. Means and 95% HPD of age estimates were obtained from the combined outputs using Tracer. The results were visualized using FigTree v1.4.2 [76].

### Resequencing, haplotype phasing, and variation analysis

Five Pyn accessions from Jeju Island, Korea, four “Yoshino cherry” accessions (Pxy) from Tokyo, Japan, and Washington, DC, USA, and seven accessions of closely related species of Pyn, including Pjj, Pjq, Ppa, and Psa, were selected for resequencing analysis (Additional file 11: Table S10). gDNA was extracted from the leaves of each accession as described above. Sequencing was performed by Illumina MiSeq, NextSeq or HiSeq (Illumina, San Diego, CA, USA) PE sequencing of libraries with 500-bp inserts according to the manufacturer’s protocol. At least 7.5× coverage of sequences was generated for each accession. All reads were preprocessed as mentioned above for quality. The genome size of each plant was calculated using the filtered reads as described above based on K-mer 17. For variome analysis, the filtered reads were aligned to the draft Pyn assembly using BWA-MEM 0.7.12 software [77] with a parameter of M. Duplicate alignments were removed using the MarkDuplicates module in the Picard 2.2.4 package [78]. Realignment around short InDels and SNP genotyping were performed using RealignerTargetCreator, IndelRealigner, and HaplotypeCaller modules in GATK 3.7 software [79]. Annotation of SNPs and InDels was performed using SnpEff 4.3p software [80]. For haplotype phasing of SNPs, SNP positions with a minimum depth of two reads were considered. BEDTools 2.25 software [81] was used to compute the alignment coverage of maternal (Ppa) and paternal (Pjj) reads to the reference sequences. To determine the phased haplotype of SNPs and to impute missing genotype calls for the draft genome sequences, BEAGLE v4.0 software [82] was used under default parameters. For further analysis of genic region, genes were phased into one of the parental types if a gene was aligned only by reads from one parent species or had at least twofold as many supports for SNPs by reads of one parental species. Otherwise, genes were defined as common type. MDS for two dimensions based on a pairwise distance matrix between two different genotypes was performed by PLINK software [83] with the parameter of −genome, −cluster, −mds-plot 2. The MDS plots were drawn using R script. MEGA7 [71] was used to draw an ML tree based on a pairwise distance matrix of SNPs, which was calculated by counting the total number of different alleles between genotypes.

## Additional files

Additional file 1: **Figure S1.** K-mer plots of wild *P. yedoensis* (Pyn) and “Yoshino cherry” (Pxy) accessions. **Figure S2.** A workflow of genome assembly and annotation. **Figure S3.** Chromosomal comparison of the gene-phased genome assembly of wild *P. yedoensis* (Pyn) with the *P. avium* (Pa) and *P. mume* (Pm) genomes. **Figure S4.** A maximum likelihood tree of S-locus genes showing the phylogenetic relationship among the S haplotypes. (DOCX 670 kb)

Additional file 2: **Table S1.** Statistics of genome sequence data of wild *P. yedoensis* (Pyn-Jeju2) used in this study. (XLSX 11 kb)

Additional file 3: **Table S2.** Statistics of transcriptome sequence data of wild *P. yedoensis* (Pyn-Jeju2) used in this study. (XLSX 11 kb)

Additional file 4: **Table S3.** Evaluation of gene space coverage of the wild *P. yedoensis* genome using transcriptome unigenes. (XLSX 10 kb)

Additional file 5: **Table S4.** Summary of repetitive sequences identified in the draft genome of wild *P. yedoensis. (XLSX 12 kb)*

Additional file 6: **Table S5.** Statistics of gene models predicted from the draft genome of wild *P. yedoensis*. (XLSX 10 kb)

Additional file 7: **Table S6.** Annotation statistics of the wild *P. yedoensis* gene set. (XLSX 10 kb)

Additional file 8: **Table S7.** Summary of alternative splicing events identified in protein-coding genes. (XLSX 10 kb)

Additional file 9: **Table S8.** Coverage of individual chromosomes of peach (Pp), sweet cherry (Pa), and Chinese plum (Pm) showing synteny with the counterpart of wild *P. yedoensis* (Pyn) genome. (XLSX 11 kb)

Additional file 10: **Table S9.** Over- or under-represented gene families in the wild *P. yedoensis* genome compared to the *P. avium*, *P. mume*, and *P. persica* genomes. (XLSX 14 kb)

Additional file 11: **Table S10.** Summary of accessions and Illumina short-read data used in whole-genome resequencing analysis. (XLSX 11 kb)

Additional file 12: **Table S11.** Comparison of the chloroplast genomes between *Prunus* accessions. (XLSX 10 kb)

## Abbreviations

bp: Base pair
CDS: Coding sequence
CTAB: Cetyl trimethylammonium bromide
DEG: Differentially expressed gene
FPKM: Fragments per kilobase million
Fv: *Fragaria vesca*
Gb: Gigabase
gDNA: Genomic DNA
GSI: Gametophytic self-incompatibility
HPD: Higher posterior density
InDel: Insertion or deletion
Kb: Kilobase
K-mer: Substring of length K
Ks: Synonymous substitution rate
Mb: Megabase
MDS: Multidimensional scaling
ML: Maximum likelihood
MP: Mate-paired
mRNA-seq: Messenger RNA sequencing
Mt: *Medicago truncatula*
Mxd: *Malus* х *domestica*
Mya: Million years ago
NCBI: National Center for Biotechnology Information
NGS: Next-generation sequencing
PCR: Polymerase chain reaction
PE: Paired end
Pjj: *P. jamasakura* var. *jamasakura*
Pjq: *P. jamasakura* var. *quelpaertensis*
Ppa: *P. pendula* f. *ascendens*
Psa: *P. sargentii*
Pxy: *P.* х *yedoensis*
Pyn: *P. yedoensis* var. *nudiflora*
SFB: S haplotype-specific F-box protein
SNP: Single nucleotide polymorphism
S-RNase: S-locus ribonuclease
Ti: Transition
Tv: Transversion
