# Supplementary material for: Draft genome sequence of wild Prunus yedoensis reveals massive inter-specific hybridization between sympatric flowering cherries
Source: Genome Biol. 2018 Sep 4;19:127. doi: 10.1186/s13059-018-1497-y (PMC6124018; doi:10.1186/s13059-018-1497-y)
Supplement: Supplementary file 1 — Figure S1. K-mer plots of wild P. yedoensis (Pyn) and “Yoshino cherry” (Pxy) accessions. Figure S2. A workflow of genome assembly and annotation. Figure S3. Chromosomal comparison of the gene-phased genome assembly of wild P. yedoensis (Pyn) with the P. avium (Pa) and P. mume (Pm) genomes. Figure S4. A maximum likelihood tree of S-locus genes showing the phylogenetic relationship among the S haplotypes. (DOCX 670 kb) [file 13059_2018_1497_MOESM1_ESM.docx]

Fig. S1. K-mer plots of wild *P. yedoensis* (Pyn) and ‘Yoshino cherry’ (Pxy) accessions. The volumes of Illumina K-mer (K=17 mer, Y axes) are plotted against the frequency where they occur (X axes). Gray line, heterozygous peak; black line, homozygous peak. Taxon ID and sequence reads used in this analysis are presented in Additional file 11: Table S10.


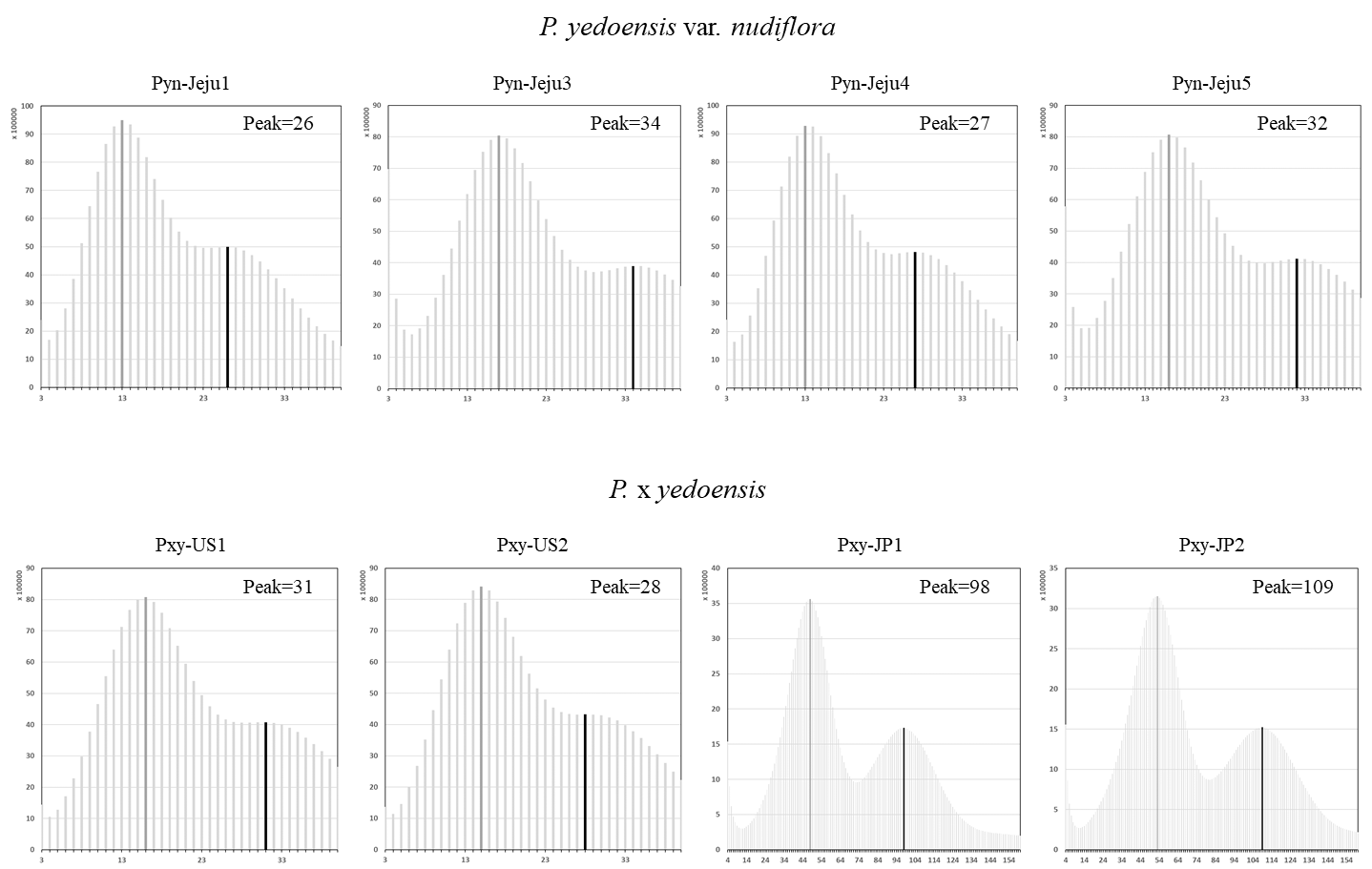


Fig. S2. A workflow of genome assembly and annotation. See Methods section for more details.


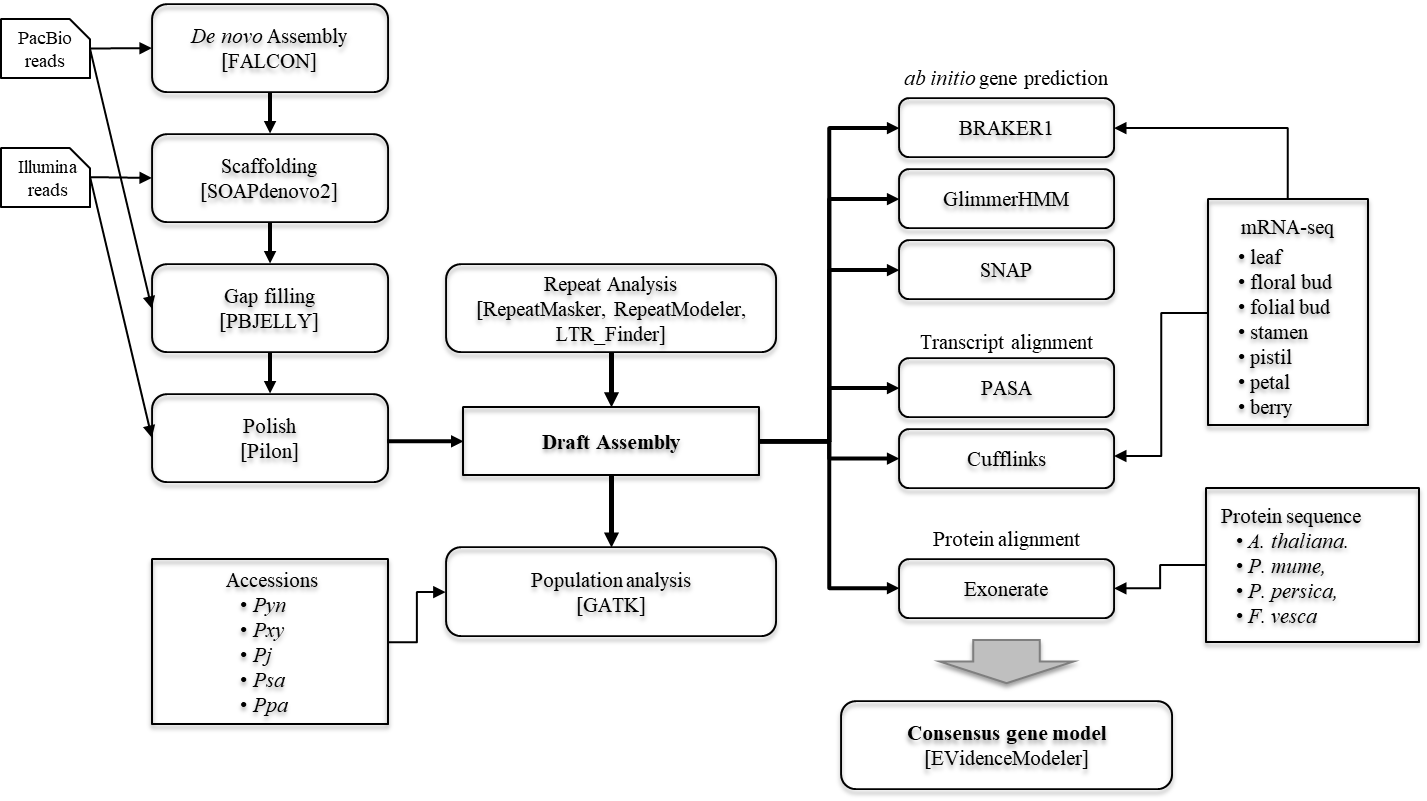


Fig. S3. Chromosomal comparison of the gene-phased genome assembly of wild *P. yedoensis* (Pyn) with the *P. avium* (Pa) and *P. mume* (Pm) genomes. Colored dots or lines represent maternal-phased genes (red), paternal-phased genes (blue), or common genes (gray).

**
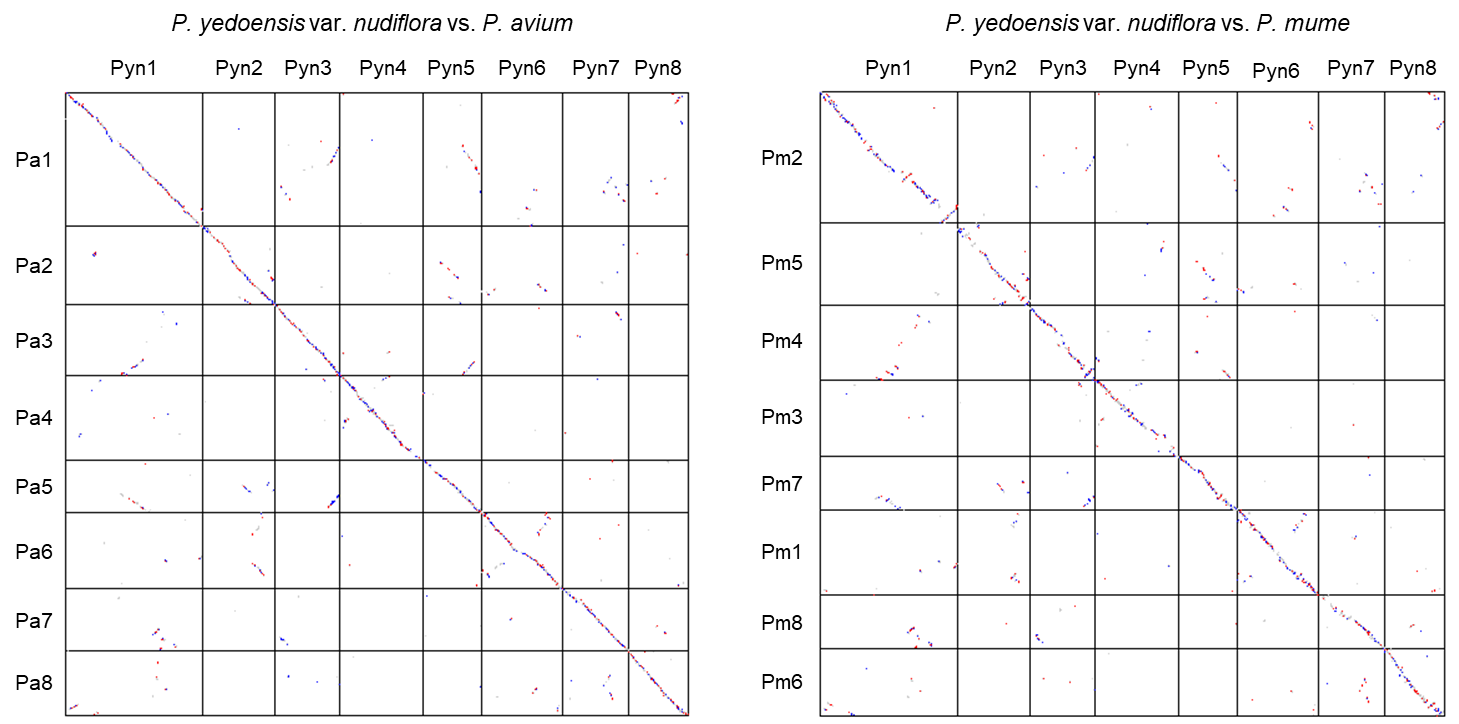
**

Fig. S4. A maximum likelihood tree of S-locus genes showing the phylogenetic relationship among the S-haplotypes. The coding sequences of *S-RNase* and *SFB* genes in S-locus regions of 12 *Prunus* accessions distributed in a sympatric natural habitat were aligned using MAFFT multiple alignment program. The trees were constructed using MEGA7 with bootstrap analysis with 1,000 replicates. *Arabidopsis thaliana* ribonuclease 3 (NP_564264, AtRNS3) and F-box protein (At3g16210) genes were used as outgroup.


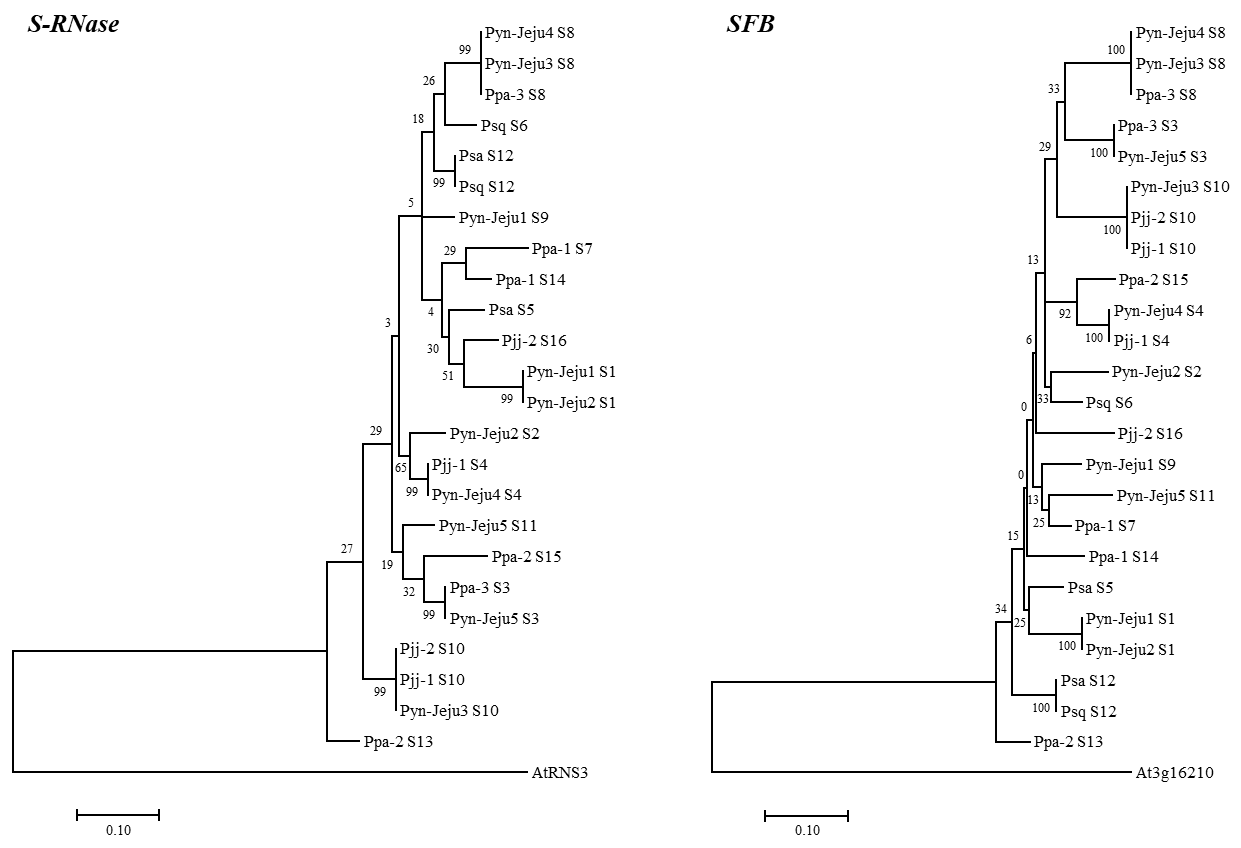


Pyn-Jeju4 S8

Pyn-Jeju3 S8

Ppa-3 S8

Ppa-3 S3

Pyn-Jeju5 S3

Pyn-Jeju3 S10

Pjj-2 S10

Pjj-1 S10

Ppa-2 S15

Pyn-Jeju4 S4

Pjj-1 S4

Pyn-Jeju2 S2

Psq S6

Pjj-2 S16

Pyn-Jeju1 S9

Pyn-Jeju5 S11

Ppa-1 S7

Ppa-1 S14

Psa S5

Pyn-Jeju1 S1

Pyn-Jeju2 S1

Psa S12

Psq S12

Ppa-2 S13

At3g16210

100

100

100

100

100

100

92

29

25

6

13

0

25

34

15

0

33

13

33

0.10

***SFB***
